# Early pregnancy ultrasound measurements and prediction of first trimester pregnancy loss: A logistic model

**DOI:** 10.1038/s41598-020-58114-3

**Published:** 2020-01-31

**Authors:** Laura Detti, Ludwig Francillon, Mary E. Christiansen, Irene Peregrin-Alvarez, Patricia J. Goedecke, Zoran Bursac, Robert A. Roman

**Affiliations:** 1 University of Tennessee Health Science Center, Division of Reproductive Endocrinology and Infertility, Department of Obstetrics and Gynecology, Memphis, Tennessee USA; 2 Cleveland Clinic, Women’s Health Institute, Department of Obstetrics and Gynecology, Cleveland, Ohio USA; 3 University of Tennessee Health Science Center, Division of Biostatistics, Department of Preventive Medicine, Memphis, Tennessee USA; 4 Florida International University, Department of Biostatistics, Miami, Florida USA

**Keywords:** Computational biology and bioinformatics, Outcomes research

## Abstract

Our objective was to prospectively validate the use of gestational sac (GS), yolk sac (YS) diameter, crown-rump length (CRL), and embryonal heart rate (HR) dimensions to identify early pregnancy loss. This was a prospective cohort study of first trimester pregnancies. GS and YS diameter, CRL, and HR measurements were serially obtained in singleton and twin pregnancies from 6 through 10 weeks’ gestation. Non-parametric tests and logistic regression models were used for comparisons of distributions and testing of associations. A total of 252 patients were included, of which 199 were singleton pregnancies, 51 were twins, and 2 were triplets (304 total fetuses). Fifty-two patients had 61 losses. We built nomograms with the changes of the parameters evaluated in ongoing, as well as in pregnancy loss. In the pregnancies which failed, all the parameters showed significant changes, with different temporal onsets: GS and YS were the first to become abnormal, deviating from normality as early as 6 weeks’ gestation (OR 0.01, 95% CI 0.0–0.09, and OR 3.36, 95% CI 1.53–7.34, respectively), followed by changes in HR, and CRL, which became evident at 7 and 8 weeks (OR 0.96, 95% CI 0.92–1.0, and OR 0.59, 95% CI 0.48–0.73, respectively). Our observations showed that, after 5 complete weeks’ gestation, a small GS and a large YS reliably predicted pregnancy loss. The YS reliably identified the occurrence of a miscarriage at least 7 days prior its occurrence. CRL and HR became abnormal at a later time in pregnancy and closer to the event. These findings have important implications for patient counseling and care planning, as well as a potential bearing on cost effectiveness within early pregnancy care.

## Introduction

Early pregnancy loss - also known as pregnancy loss, fetal demise, miscarriage, or spontaneous abortion - is defined as a “nonviable, intrauterine pregnancy with either an empty gestational sac or a gestational sac containing an embryo or fetus without fetal heart activity prior to 12 weeks and 6 days of gestation”^1^. It is the most common complication of early pregnancy, affecting about 30% of pregnancies following assisted reproduction and 10% of spontaneously conceived pregnancies^2–4^. The difference is explained by a later diagnosis of spontaneous pregnancy versus assisted reproduction pregnancy, and an early loss is easily overlooked. In fact, vaginal bleeding - a common sign of early pregnancy loss - can be confused with delayed menses and the loss remains unrecognized. The most common cause of a first trimester pregnancy loss is embryonal genetic abnormalities, which occurs in more than 50% of the cases, with aneuploidy being the most frequent abnormality^5,6^.

Multiple serologic and ultrasound markers have been investigated to identify pregnancies destined to be lost^7,8^. However, serologic markers are unspecific and can help only after a pregnancy loss has already been diagnosed. Transvaginal ultrasound (TVUS) provides high-resolution images, low inter-observer variability with high reliability, and is typically used to make diagnosis of intrauterine pregnancy and to follow up with its development^9^. Gestational sac (GS), yolk sac (YS), crown-rump length (CRL), and heart rate (HR) are the parameters measured to evaluate early pregnancy. Deviations in the ultrasound parameters have been alternatively investigated to predict first trimester pregnancy loss. The amniotic sac, which becomes visible at the beginning of the 7^th^ week of gestation, is normally not contemplated in the prediction models, however it assists in dating a pregnancy correctly.

Logistic models have been used to assess predictability of pregnancy loss using ultrasound parameters as dependable variables. One model including 566 gravidas, 7.9% of whom had an early pregnancy, identified HR and CRL as the most significant parameters to predict a pregnancy loss, together with maternal age and vaginal bleeding^8^. Another one evaluated pregnancies achieved by *in vitro* fertilization and found that multiple variables including maternal age, duration of infertility, GS diameter, CRL, HR, and YS, predicted an early pregnancy loss better than each individual parameters^10^. However, the model did not include an exact gestational age and included variables, such as maternal age, which alone is a well-established risk factor for first trimester pregnancy loss^11^. Another model reported that a CRL, GS, and HR, below the 5^th^ percentile, and a YS diameter above the 95^th^ percentile would predict early pregnancy loss (odds ratio 1.04). However, a normal YS would not decrease the risk of pregnancy loss when the other parameters were abnormal^12^. A systematic review evaluated sensitivities and specificities of the ultrasound parameters and found that HR ≤ 110 beats per minute (BPM) was the most reliable model to predict a subsequent pregnancy loss, with a sensitivity of 68.4%, a specificity of 97.8%, a positive likelihood ratio of 31.7 (95% confidence interval 12.8–78.8), and a negative likelihood ratio of 0.32 (95% confidence interval 0.16–0.65). In pregnancies with vaginal bleeding, in addition to an HR ≤ 110 BPM, prediction of an early loss was higher^13^. All the discussed early pregnancy ultrasound markers have been alternatively found to predict first trimester loss, however they have never been evaluated longitudinally, and only one ultrasound per patient was included in the analyses^10,13–15^.

The yolk sac has been individually studied as a marker of pregnancy loss. Being identified at approximately 5 weeks of gestation and gradually increasing in size in a linear fashion until 10 weeks of gestation, the YS is the first identifiable structure via transvaginal ultrasonography within the GS. In particular, a YS larger than 6.0 mm at any gestational age was associated with early loss, while an abnormal shape would not carry an ominous prognosis^15,16^. Our group established a nomogram of YS growth from its first appearance until 10 weeks of gestation and found that deviations from the typical growth pattern were associated with a pregnancy loss^17^.

Previous studies were cross sectional and provided estimates for pregnancy loss that were based on a combination of ultrasound, as well as serologic and demographic markers. The aim of this study was to estimate a risk of first trimester pregnancy loss based solely on ultrasound findings. Thus, we longitudinally evaluated the GS, YS, CRL, and HR changes in singleton and multiple pregnancies with definite conception dates in order to build nomograms of their changes up to 10 weeks of gestation. In addition, we wanted to identify which parameters were the first and most reliable to predict a pregnancy loss in singleton and multiple pregnancies. Our hypothesis was that different markers would sequentially become abnormal at different embryonal stages, when a pregnancy is destined to be lost.

## Materials and Methods

**Figure 1:**
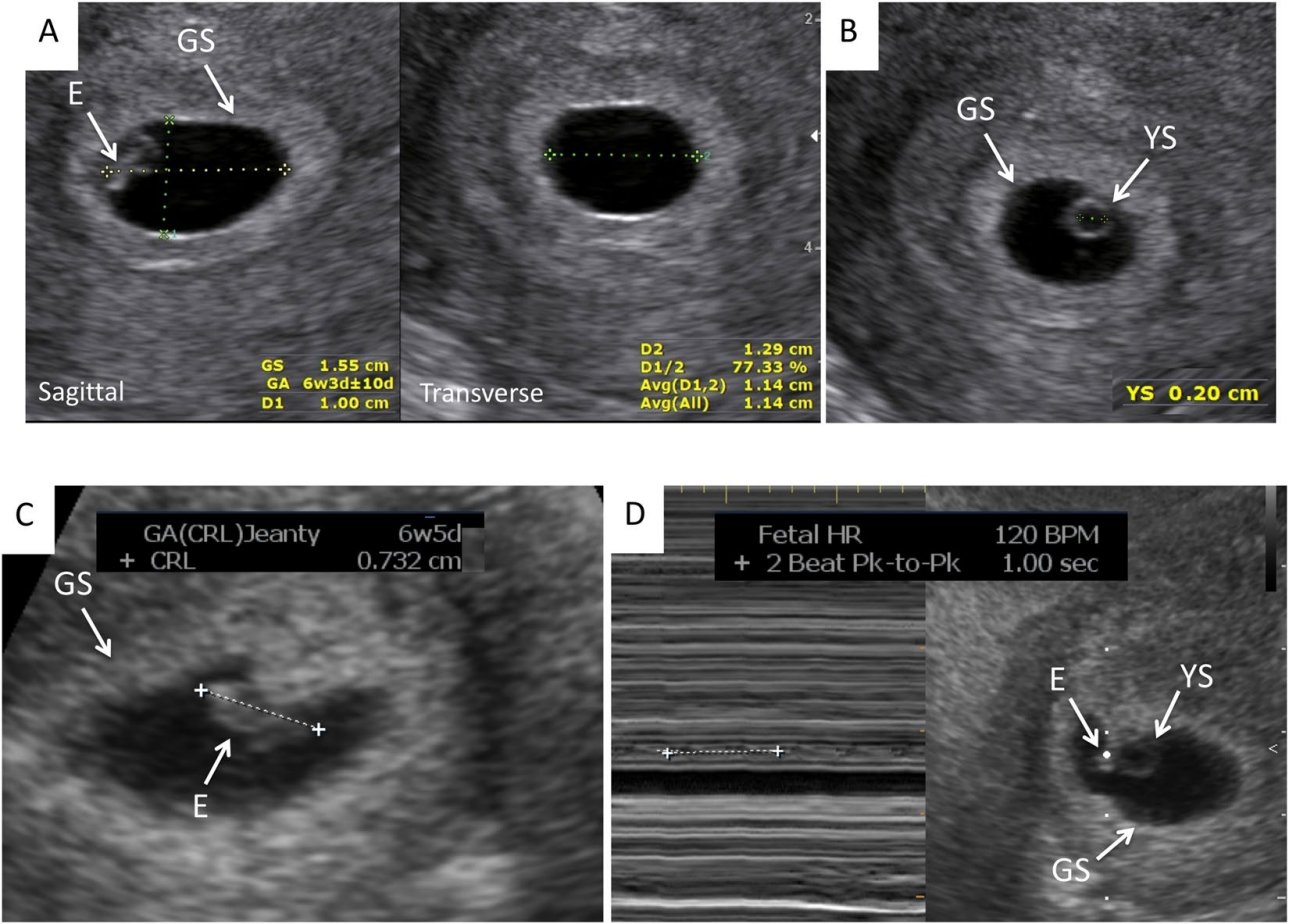
Correct cursor position for the measurement of the parameters under investigation: (**A**) Gestational sac (GS); (**B**). Yolk sac (YS); (**C**). Crown-rump length (CRL); (**D**). Heart Rate (HR). E = Embryo.

This was a prospective cohort study. The conduct of this study was approved by the University of Tennessee Health Science Center Human Investigation Committee and the study is currently registered at ClinicalTrials.gov (NCT02429336). All methods were performed in accordance with the relevant guidelines and regulations. All patients gave informed, written consent to participate in the study. The patients in our study were all evaluated and treated for infertility and had known conception dates. The mode of conception included spontaneous, after superovulation with clomiphene citrate or letrozole with, or without, intrauterine insemination (IUI), and *in vitro* fertilization (IVF) after superovulation with gonadotropins. The GS and YS diameter, CRL, and HR measurements were obtained with 2-D transvaginal ultrasound in singleton, and multiple pregnancies followed from 6 through 11 weeks’ gestation. For the scans we used two ultrasound machines: Philips XD11 with a 7.5 MHz transvaginal probe and a Samsung UGEO WS80A 3-D with a 7.5 MHz transvaginal probe. All measurements were obtained on a magnified, frozen section (sagittal and/or transverse) of the parameter to be evaluated. Measurements of the GS were obtained in three dimensions (length, height, width), the YS diameter was measured from one inner rim to the opposite inner rim. If not spherical, the three dimensions were measured and averaged. The CRL was measured once and the FHR was measured once with M mode. Both parameters’ measurements were repeated in different sections if the first measurement did not meet the expected value for gestational age. The ultrasound machine provides the expected gestational age for each variable measured based on standardized algorithms, except for YS. For YS, we used the previously established nomogram of YS growth from 5 until 10 weeks of gestation^17^. Figure 1 shows the correct cursor position for the measurement of the parameters under investigation. The GS largest diameter was measured in the three orthogonal planes and averaged (Fig. 1A). The YS largest diameter was measured placing the calipers at the inner rim of the organ. CRL was measured placing the calipers in the most cephalad and most caudal extremities of the embryo’s longitudinal image. HR was automatically calculated by the machine, averaging the distance between one, or two, systolic spikes.

All the clinic patients with a positive pregnancy test were invited to come to the clinic for an initial transvaginal ultrasound at 5–6 weeks of gestation, depending of the patient’s history. All patients presenting between January 2014 and December 2017 were included in the study. Most patients had weekly ultrasounds from 5 to 11 weeks of gestation, for an average of 4 scans per patient. To maintain consistency, a single examiner performed all of the sonographic exams of this study. If at the initial scan the embryo had no cardiac activity, all parameters were measured to confirm the gestational age and a second scan was performed one week later to confirm the outcome. If no gestational sac was present, and an ectopic pregnancy was excluded, or the mean gestational sac diameter measured more than 20 mm without a visible YS or embryonal pole, these were classified as a nonviable pregnancy and were excluded from the analyses. In this cohort, there were no pregnancies that ended in elective termination.

Recurrent pregnancy loss was defined as two or more first trimester pregnancy losses^18^. All pregnant women were discharged from the Reproductive Medicine clinic between 10 and 11 weeks of gestation. Pregnancy outcome was determined through the evaluation of hospital medical records.

### Statistical analysis

Variables, even if continuous, were expressed as Median and quartiles (Q1, Q3) because the Median is not skewed so much by a small proportion of extremely large or small values and it is more representative of a typical value. All analyses were performed using SAS/STAT V14.1 (Cary, North Carolina, USA). Mann-Whitney U test and Pearson correlations were used for comparisons between the ongoing pregnancy and pregnancy loss groups (Table 1). The GS and YS diameters, CRL and FHR were plotted relatively to gestational age. Descriptive statistics including medians and quartiles for GS, YS, CRL and HR for each gestational week were calculated by pregnancy loss status. If a patient had a loss before and a continuing pregnancy after, she was allocated to the group which identified the outcome at the time of her pregnancy. If the patient had twins, or triplets, with one or two losses within the same pregnancy (=vanishing twin), to balance the results she was allocated to both groups. We applied Wilcoxon 2-sample tests by gestational week for univariate comparison of distributions/medians for GS, YS, CRL, and HR, between the pregnancies that were lost and those that were not. For YS, we also performed a median split analysis using Wilcoxon 2-sample tests by gestational week both above and below the YS median, to compare the YS medians by pregnancy loss status. We calculated the gestational age in weeks, rather than in days, because it is the standard method to measure the gestational age in clinical practice. We extended the univariate analysis into multivariate logistic regression models in order to retain multiple significant predictors of pregnancy loss by gestational week. Non-significant variables were omitted from the model unless contributing to the overall fit of the model. We estimated optimal sensitivity and specificity of each model along with area under the receiver operating characteristic curve (AUC). All associations were considered significant at alpha level 0.05.

**Table 1:** Demographics of the patient population divided by pregnancies that resulted in a first trimester loss and those that progressed beyond the first trimester (continuing pregnancy).

Demographics	First Trimester Pregnancy Loss N = 52	Continuing Pregnancy N = 243	p-value
Age (years) [Median (Q1, Q3)]	34.0 (31.0, 36.3)	32.0 (29.0, 35.0)	0.067^a^
Gravidity (N) [Median (Q1, Q3)]	2.0 (1.0, 3.0)	2.0 (1.0, 3.0)	0.440^a^
Parity (N) [Median (Q1, Q3)]	0 (0, 1.0)	0 (0, 1.0)	0.324^a^
BMI (kg/m^2^) [Median (Q1, Q3)]	26.1 (22.0, 30.5)	25.0 (22.0, 31.0)	0.785^a^
Mode of conception* (percentage)			
*Spontaneous*	22 (42.3%)	66 (31.6%)	0.189^b^
*Ovulation induction (OI)*	8 (15.4%)	27 (12.9%)	0.399^b^
*OI + Intrauterine Insemination*	6 (11.5%)	27 (12.9%)	0.060^b^
*In vitro Fertilization*	16 (30.7%)	89 (42.6%)	0.081^b^
Clinical Condition** (percentage)			
*Smoking*	2 (3.8%)	14 (6.7%)	0.440^b^
*PCOS*	35 (67.3%)	154 (73.7%)	0.357^b^
*Glucose Intolerance*	6 (11.5%)	29 (13.9%)	0.658^b^
*Hypothyroidism*	8 (15.4%)	34 (16.3%)	0.877^b^
*Hypertension*	1 (1.9%)	4 (1.9%)	0.997^b^
*Autoimmune disease*	2 (3.8%)	16 (7.7%)	0.429^b^
*Uterine Subseptation*	12 (23.1%)	30 (14.4%)	0.126^b^
*Uterine Fibroids*	6 (11.5%)	33 (15.8%)	0.442^b^
*Endometriosis*	3 (5.8%)	17 (8.1%)	0.566^b^

^a^Mann-Whitney U test; ^b^Pearson Chi-Square.

^*^In twin, or triplet, pregnancies whit one, or two, miscarried embryos and one viable embryo, the same patient was counted in both groups.

**Pregnancies in both groups occurred after corrections of the underlying conditions. PCOS was diagnosed using the Rotterdam criteria^17^; Glucose intolerance was defined as hemoglobin A1c ≥ 5.7%; Hypothyroidism was defined by a TSH ≥ 2.5 mIU/l; Hypertension was defined as blood pressure ≥140/90; Autoimmune disease encompassed systemic Lupus Erythematosus, Lupus Anticoagulant, Anticardiolipin antibodies, AntiPhosphatidil antibodies; Uterine subseptations were treated when measuring ≥5.9 mm.

## Results

Of the 252 pregnancies included in this study, 199 (78.9%) were singleton pregnancies, 51 (20.2%) were twins (3 of which were monochorionic and 48 dichorionic), and 2 (0.008%) were triplets (monochorionic twins plus a singleton; both pregnancies spontaneously reduced to singleton at 7 weeks of gestation), for a total of 304 embryos longitudinally studied (one of the twin pregnancies had an empty GS, which was excluded from the calculations). Thirty-six of 252 pregnancies (14.3%) had a first ultrasound between 4–5 weeks of gestation because of history of ectopic pregnancy, recurrent pregnancy loss, pelvic pain, or vaginal bleeding. For 21 patients, no delivery information was available, however they were lost to follow-up after their third-trimester ultrasound and were included in the analyses in the group of patients who had a continuing pregnancy.

Sixty-one of 304 (20%) embryos, in 52 pregnancies, were lost: 20/61 (32.8%) in twin, or triplet, pregnancies and 41/61 (67.2%) in singleton pregnancies. The remaining 243 embryos progressed beyond the first trimester. Thirty-three of 61 embryos (54.1%) were already lost at the time of the initial ultrasound, of which 19 (31.1%) at 4–5 weeks and 14 (22.9%) at 6 weeks of gestation. Of the pregnancies that were lost, only 5 had vaginal bleeding as the initial sign of pregnancy failure, all in singleton pregnancies. Neither of the twin pregnancies with a vanishing or demised twin underwent genetic analysis. Of the 61 pregnancy losses, 18 singleton and 1 twin pregnancies underwent microarray analysis for genetic abnormality: results were inconclusive in two instances, and unknown in one. Twelve of 17 (70.6%) showed chromosomal abnormalities: 4 were trisomy 21, 2 were trisomy 16, 2 were trisomy 22, 2 were triploid, and 2 were complex genetic abnormalities. All embryos had a YS diameter larger than the median in continuing pregnancies pregnancies. Five of 17 (29.4%) were normal karyotypes and all embryos had smaller or similar YS diameter compared to the median in continuing pregnancies.

Table 1 reports the demographics and the clinical characteristics of patients that had a first trimester pregnancy loss (N = 52, 61 lost embryos) and those who continued the pregnancy beyond the first trimester (N = 209, 243 fetuses). Variable measurements in the continuing pregnancy group conformed to the expected value by gestational age calculated by the ultrasound machine and were considered the normal cut-offs at each gestational age. Patients with twin/triplet pregnancies who lost one, or two, embryos, but continued the pregnancy with the remaining fetus/es (13/51 twins and 2/2 triplets) were allocated to both groups. In this way, maternal characteristics would have the same statistical weight in the two groups. There was no difference in age, BMI, gravidity, parity, mode of conception, and clinical history (all not significant to <0.05). Spontaneous conception was the most common mode of conception in the pregnancy loss group, while IVF was the most common in the continuing pregnancy group; polycystic ovary syndrome was the most common preexisting clinical condition in both groups, followed by uterine subseptations. Seventy-one patients had had one, or two, pregnancy losses prior to the index pregnancy. Twenty-six patients had a diagnosis of recurrent pregnancy loss (6 in the pregnancy loss, and 20 in the continuing pregnancy group). Two twin pregnancies were delivered at 26 weeks of gestation: one for preterm delivery and the other for severe preeclampsia in a 44 year-old woman who had conceived through egg donation. One singleton pregnancy was included in the continuing pregnancy group even though it was complicated by fetal demise at 24 weeks from a tight nuchal cord. The fetus had a normal male karyotype, 46, XY. Fifty-eight additional fetuses were delivered between 32 and 36 6/7 weeks of gestation, mostly from preterm delivery of twin pregnancy (45 fetuses in twin pregnancies, and 13 fetuses in singleton pregnancies), and the remaining 180 fetuses were delivered at term (≥37 weeks of gestation). None of the neonates had genetic abnormalities. All the investigated parameters became significantly different in pregnancies destined to be lost, but with a different chronology. Aside from the GS dimensions in monochorionic twins, there was no difference in dimension of any other parameters in singleton versus multiple pregnancies.

**Figure 2:**
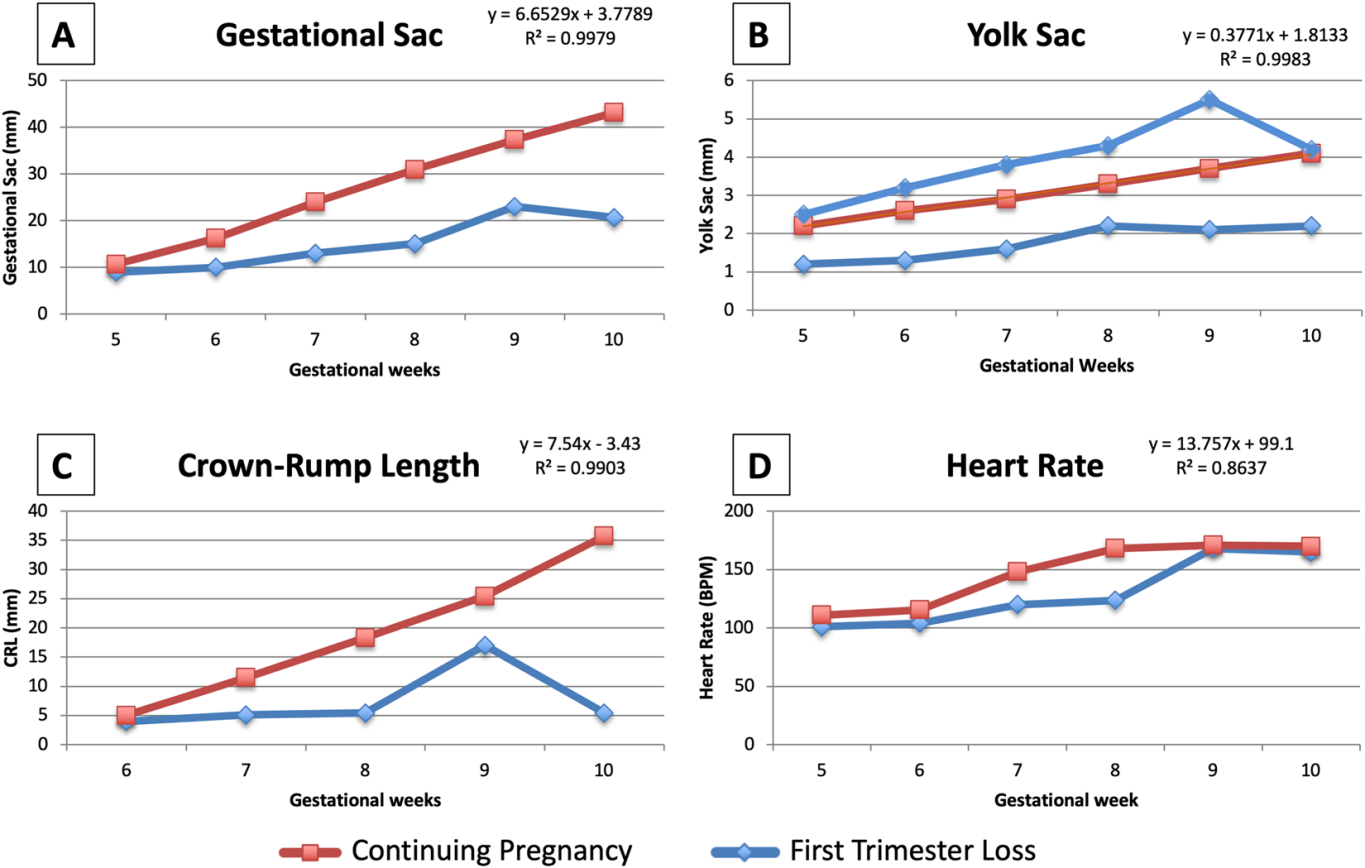
Median measurements of the parameters under investigation in the two groups, pregnancy loss and continuing pregnancy, plotted against the gestational age: (**A**). Gestational sac (GS); (**B**). Yolk sac (YS); (**C**). Crown-rump length (CRL); (**D**). Heart Rate (HR).

Figure 2 shows the median GS, YS, CRL, and HR measurements at the gestational ages under investigation in the two groups, pregnancy loss and continuing pregnancy. Variable measurements in the continuing pregnancy group conformed to the expected gestational age by the ultrasound machine and were considered the normal cut-offs at each gestational age. The GS diameter grew 6.65 mm per week (R^2^ = 0.9979) in pregnancies that continued beyond the first trimester and it was smaller in pregnancies destined to be lost, however the difference was not significant until 8 weeks of pregnancy, when the median diameter of the gestational sac was 15 mm (IQR 12, 21 mm) in pregnancy losses and 31 mm (28, 35 mm) in continuing pregnancies (p < 0.001, Fig. 2A, Table 2). The YS grew 0.38 mm per week (R^2^ = 0.9983) in pregnancies that continued beyond the first trimester. In pregnancies destined to be lost, the YS was either smaller, or larger, than in continuing pregnancies starting at 5 weeks of gestation, and maintained the trend until the pregnancy loss was diagnosed (Fig. 2B, Table 2). The CRL grew 7.54 mm (R^2^ = 0.9903) per week and was significantly larger in the continuing pregnancy than in the pregnancy loss group from 6 through 10 weeks (Fig. 2C, Table 2). HR increased from 5 weeks of gestation and became significantly different in the two groups between 7 and 8 weeks of gestation, when it increased by 13 BPM in the continuing pregnancy, versus increasing 3 BPM in the pregnancy loss group. HR still fit a linear relationship with gestational age, increasing by 13.76 BPM per week, even though with a lower R^2^ of 0.8637.

**Table 2:** Univariate analysis of all the evaluated parameters stratified by pregnancy loss status for each week of gestation.

Week	Gestational	Variable	Continuing Pregnancy		Pregnancy Loss		
	days		N	Median (Q1, Q3)	N	Median (Q1, Q3)	p value
5	35–41		28	2.2 (2.1, 2.5)	9	2.3 (1.6, 2.6)	0.9148
6	42–48		149	2.6 (2.3, 2.9)	19	3.0 (2.2, 3.7)	0.1641
7	49–55	Yolk sac	162	2.9 (2.6, 3.3)	30	3.7 (3.0, 5.0)	<0.0001
8	56–62	(mm)	150	3.3 (3.0, 3.7)	24	4.1 (3.1, 5.2)	0.0040
9	63–69		141	3.7 (3.4, 4.0)	11	3.5 (2.1, 5.5)	0.8307
10	70–76		117	4.1 (3.7, 4.5)	5	2.2 (2.2, 3.2)	0.0064
5	35–41		31	11 (7, 13)	11	9.0 (6, 11)	0.2236
6	42–48		152	16 (13, 19)	25	10 (7, 13)	<0.0001
7	49–55	Gestational sac	159	24 (21, 27)	35	13 (11, 21)	<0.0001
8	56–62	(mm)	146	31 (28, 35)	30	15 (12, 21)	<0.0001
9	63–69		140	37 (34, 42)	16	23 (19, 27)	<0.0001
10	70–76		122	43 (39, 48)	11	21 (19, 22)	0.0003
5	35–41		21	2.5 (1.8, 4.3)	7	2.3 (1.5, 3.2)	0.3952
6	42–48		153	5 (4.2, 6.4)	19	4.0 (2.7, 4.4)	<0.0001
7	49–55	Crown-Rump	163	11.5 (9.6, 13.6)	30	5.1 (3.6, 8.4)	<0.0001
8	56–62	Length (mm)	152	18.3 (16.7, 20.0)	22	5.4 (3.9, 12.1)	<0.0001
9	63–69		145	25.4 (23.2, 28.6)	12	17.0 (8.1, 25.0)	0.0013
10	70–76		126	35.8 (33.0, 38.1)	5	5.4 (5.2, 15.0)	0.0082
5	35–41		14	111 (89, 121)	3	101 (94, 115)	0.6583
6	42–48		150	116 (110, 123)	12	104 (98, 126)	0.1659
7	49–55	Heart Rate	162	148 (134, 158)	15	120 (101, 150)	0.0019
8	56–62	(BPM)	152	168 (164, 174)	6	124 (84, 162)	0.0221
9	63–69		145	171 (166, 175)	2	168 (158, 178)	0.9198
10	70–76		128	170 (164, 174)	1	165	0.5005

Univariate comparisons are reported in Table 2. In early gestational weeks (weeks 7 and 8), a larger median YS was associated with an increased risk of pregnancy loss, whereas in week 10, a smaller median YS was associated with an increased risk of pregnancy loss. Starting at 6 weeks of gestation up to 10 complete weeks, smaller GS and CRL were associated with a subsequent pregnancy loss (p < 0.01 for all). A slower HR was predictive of a subsequent pregnancy loss at 7 through 8 weeks of pregnancy (p < 0.05 for both), however, this did not hold true for a slower HR occurred prior to 7 weeks or after 8 weeks.

**Figure 3:**
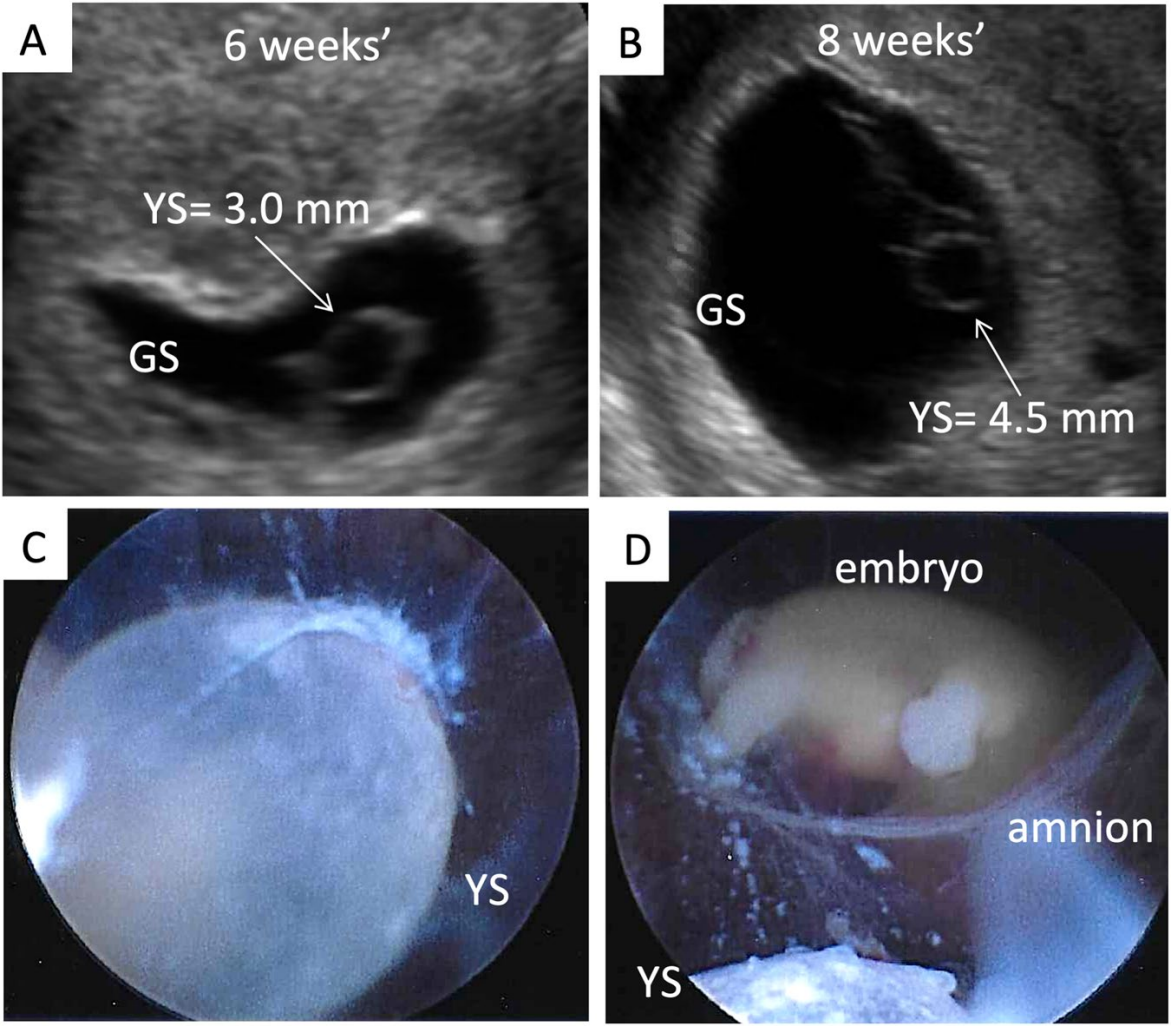
(**A**) Ultrasound and hysteroscopic images of the yolk sac in a partial mole pregnancy (Karyotype: 69, XXY at microarray analysis). (**A**) Ultrasound picture showing an enlarged yolk sac at 6 weeks and 1 day of gestation; (**B**). Ultrasound picture showing an enlarged yolk sac at 8 weeks and 2 days of gestation; (**C**). Hysteroscopic view of the yolk sac at the time of pregnancy evacuation at 8 weeks and 2 days of gestation, after embryonal demise. (**D**) A portion of the yolk sac can be noted just outside of the amniotic sac, with the embryo within it, in the background. GS = gestational sac; YS = yolk sac.

This analysis further confirmed our findings when we subdivided the pregnancy loss group into those below and above the expected YS median per gestational age. In fact, a smaller YS diameter was associated with pregnancy loss at week 6 (trend), and 8–10 of gestation (p < 0.05 for all), and a larger YS diameter was associated with pregnancy loss from week 6–9 of gestation (p < 0.05 for all; Table 3). Figure 2B reports the YS diameter of pregnancy losses at different gestational ages compared to continuing pregnancies. Figure 3 shows ultrasound and hysteroscopic images of an enlarged YS in a 69, XXY pregnancy. Using the significant univariate models and after excluding 19 pregnancy losses diagnosed at the time of the initial ultrasound, 43% of the losses (18/42) could be predicted at least one week before they occurred. In particular, a smaller GS, a shorter CRL, and a larger YS, could predict 43% of the pregnancy losses 1 week in advance in 9 cases, 2 weeks in advance in 7 cases, 3 weeks in advance in 1 case, and 5 weeks in advance in 1 case. A slow embryonal HR was not amply anticipatory of a loss.

**Table 3:** Univariate comparisons for the yolk sac during the 5–10 complete gestational weeks, divided into below-, and above, the median yolk sac measurement in pregnancies that continued beyond the forst trimester.

Gestational week	Gestational days	Yolk Sac (mm)	Continuing pregnancy		Pregnancy Loss		p-value
			N	Median (Q1, Q3)	N	Median (Q1, Q3)	
5^th^	35–41	below median	15	2.1 (1.8, 2.2)	4	1.4 (1.1, 1.9)	0.1166
		above median	13	2.5 (2.4, 2.7)	5	2.6 (2.4, 3.0)	0.4778
6^th^	42–48	below median	67	2.2 (2.1, 2.4)	8	1.9 (1.3, 2.3)	0.0533
		above median	82	2.8 (2.7, 3.1)	11	3.6 (3.2, 4.6)	<0.0001
7^th^	49–55	below median	84	2.6 (2.3, 2.8)	7	2.6 (1.6, 2.8)	0.5088
		above median	78	3.3 (3.1, 3.5)	23	4.3 (3.5, 5.7)	<0.0001
8^th^	56–62	below median	74	3.0 (2.8, 3.1)	7	2.3 (2.1, 3.0)	0.0161
		above median	76	3.7 (3.4, 3.9)	17	4.5 (4.0, 5.5)	<0.0001
9^th^	63–69	below median	70	3.4 (3.1, 3.5)	6	2.7 (1.8, 3.3)	0.0473
		above median	71	4.0 (3.8, 4.4)	5	5.5 (4.5, 7.6)	0.0183
10^th^	70–76	below median	58	3.7 (3.2, 3.9)	4	2.2 (2.0, 2.7)	0.0003
		above median	59	4.5 (4.2, 4.9)	1	4.2 (4.2, 4.2)	0.5167

Wilcoxon Signed Rank test.

Results of the logistic regression models for the multivariate analysis are displayed in Table 4. These regressions were performed by gestational week, with pregnancy loss as the outcome variable. Sensitivity and specificity were estimated from the models, AUC is presented as a measure of model fit. These adjusted models largely confirm the univariate findings. A larger YS was associated with a 3–6 times increased chance of pregnancy loss from 5 through 8 weeks. At 10 weeks of gestation, instead, a larger YS was associated with a decreased chance of pregnancy loss. This is reflected in the fact that only one pregnancy loss after 10 weeks of gestation had an enlarged YS. In fact, all the losses at that gestational age showed a YS smaller than the median for pregnancies continuing beyond the first trimester. A larger GS was associated with a decreased chance of losing the pregnancy, suggesting that a smaller GS, instead, is indicative of pregnancy loss. The direction of this association was consistent over the time frame under investigation, reaching significance at, 6, 7, and 9 weeks. Similarly, a larger CRL measurement was associated with a decreased chance of pregnancy loss, again indicating that a smaller CRL at 8 and 9 weeks of gestation predicts a pregnancy loss. The models performed with reasonable predictive accuracy and goodness of fit. Sensitivity ranged from 60–86% and specificity from 78–91%.

**Table 4:** Multivariate logistic regression models with the significant predictors of pregnancy loss, and their sensitivity and specificity.

Gestational Week	Gestational days	Variables	Odds Ratio	(95% CI)	p-value	Sens. (%)	Spec. (%)	AUC	N
5^th^	35–41	Yolk Sac	<0.001	(<0.001 5.34)	0.0881	79	67	0.95	17
		Heart Rate	0.97	(0.86 1.10)	0.6482				
6^th^	42–48	Yolk Sac	3.36	(1.53 7.34)	0.0025	74	78	0.86	166
		Gestational Sac	0.01	(0.00 0.09)	<0.0001				
7^th^	49–55	Yolk Sac	6.52	(2.32 18.33)	0.0004	79	73	0.90	173
		Gestational Sac	0.10	(0.02 0.52)	0.0063				
		Heart Rate	0.96	(0.92 1.00)	0.0376				
8^th^	56–62	Yolk Sac	6.28	(1.21 32.73)	0.0291	86	83	0.92	172
		Crown-rump Length	0.59	(0.48 0.73)	<0.0001				
9^th^	63–69	Gestational Sac	0.10	(0.02 0.41)	0.0016	82	85	0.86	151
		Crown-rump Length	0.85	(0.71 1.02)	0.0806				
10^th^	70–76	Yolk Sac	0.05	(0.08 0.33)	0.0018	60	79	0.92	118
		Gestational Sac	0.57	(0.24 1.35)	0.1975				

Multivariate Logistic Regression; 95% CI = 95% Confidence Interval; Sens. = Sensitivity; Spec. = Specificity; AUC = Area Under the Curve; N = Number of cases in each group.

## Discussion

In pregnancies destined to be lost, different ultrasound markers became abnormal at least one week before the loss. We established that the GS, CRL, and YS are the first parameters to become abnormal, as early as 5 weeks of gestation, and that HR becomes abnormal at a later time and only for a brief period prior to the loss. In addition, multiple markers predict the outcome with increased sensitivity and specificity compared to each individual marker.

The pregnancy loss rate of 20% in our study was comparable to the one reported for IVF^2^, but higher than the one reported for spontaneous pregnancies (about 10%)^3,4^. However, in our cohort most losses occurred within 14 days of the missed menses, and in different circumstances where the conception date is not known, a pregnancy, and hence a pregnancy loss, would most often go unrecognized. The fact that spontaneous conception was the most common mode in the pregnancy loss group, while IVF was the most common in the continuing pregnancy group could be due to the different support of the luteal phase and early pregnancy stages, as IVF pregnancies are supported with gonadotropins prior to, and progesterone after, the day after oocyte retrieval (corresponding to the day of ovulation in spontaneous pregnancies), which change the endometrial characteristics compared with spontaneously conceived pregnancies. Polycystic ovary syndrome was the most common preexisting clinical condition in both groups; it was treated in all patients with daily metformin, 500 mg to 2000 mg. These results might help understanding the etiology of pregnancy loss in women with this condition, which in our study, seemed to be unrelated to the hyperinsulinemic status, often reported as the most probable cause of pregnancy loss^19^. However, studies powered to explore our incidental finding would be needed.

The YS appeared to be the strongest marker for the prediction of a pregnancy loss. Other studies have established YS as a reliable predictor of pregnancy outcome, however these studies are limited by their cross-sectional evaluation with only one ultrasound per patient^10,12–16^. In our study we performed multiple ultrasounds to accurately represent all gestational ages in each patient. We previously described a nomogram of YS development during the first 10 weeks of pregnancy with serial ultrasounds in pregnancies that continued beyond the first trimester^17^. We confirmed a YS linear growth of approximately 0.4 mm per week in this larger patient sample. Our findings corroborate a large cross sectional study with over 4,000 patients^20^. After 5 complete weeks of gestation, the YS reliably detects pregnancies destined to be lost, also confirmed by multivariate analysis. In pregnancies destined to fail, the YS was either smaller or larger than in pregnancies continuing beyond the first trimester. While all pregnancies with a large YS were lost within 10 weeks, some pregnancies with smaller YS were lost beyond 10 weeks of pregnancy. The etiology of a large YS is essentially unknown, however 18–66% of large YS diameters greater than 5–6 mm have been associated with abnormal karyotypes^21,22^. Our limited genetic results seem to corroborate these previous findings.

CRL is difficult to measure at 6 weeks of gestation, being subject to the sonologist’s experience and the ultrasound machine’s capabilities. Several nomograms for CRL have been developed in different countries by cross sectional studies. An international nomogram of CRL growth was recently developed for pregnancy dating, however measurement started at 9 weeks of gestation^23^. Between 6 and 10 weeks of gestation, another cross sectional study found a quadratic relationship between CRL and gestational age^20^. Our results, dating from 5 weeks of gestation, defined a linear fit of CRL growth, up to 10 weeks. CRL was a weak predictor of pregnancy loss between 6 and 8 complete weeks of gestation, however it became a stronger predictor when combined with YS or GS abnormalities. In addition, the growth lag usually preceded the event by less than one week, thus providing little time for counseling.

Many HR nomograms have been developed, and one with the largest data was by Papaioannou^20^. In this cross sectional study a cubic association between HR and gestational age was found. However those pregnancies were followed through 13 weeks of gestation, when a natural slowing in HR is observed. We described a linear relationship through 10 weeks of gestation with an excellent R^2^ value. Given the rather important variation in BPM per second, a slower HR is not a reliable tool to predict the occurrence of a pregnancy loss unless it is below 100 BPM at a gestational age greater than 6 weeks of gestation^24^. In our study, a HR slower than in continuing pregnancies was predictive of a subsequent pregnancy loss only between 7 and 8 weeks of gestation, but not prior, or after, this time. Even if highly specific of pregnancy loss when absent, HR abnormalities presented very close to the event, thus providing little time for counseling.

A major strength of our study is the advantage of a single investigator performing all the ultrasounds, thus maintaining consistency in the measurements, with small inter-observer variability. Additionally, all subjects included in the study had precisely known gestational ages further strengthening the accuracy of our results. Limitations of the study include the relatively small sample size, along with a patient population treated for infertility, which may make our results not generalizable to spontaneous conceptions. Additionally, some pregnancies were already lost at the time of the first ultrasound at 5 or 6 weeks of gestation, and we were not able calculate the interval between the measured abnormal parameter and the loss. In fact, our model was suitable mostly for pregnancies that had an ultrasound at 6 weeks and were lost at 8–9 weeks of gestation, or later. We analyzed the data ‘per week’ of gestation to reflect the standard gestational age quantification, however, we may have lost sharpness of the results as compared to analyzing the data ‘per day’ of gestation.

In conclusion, we were able to establish a statistical model using *only* early pregnancy ultrasound markers to predict a first trimester loss. GS and YS were the earliest parameters that could reliably be used as prognostic factors for pregnancy loss, as they became abnormal as early as 6 weeks of gestation with high sensitivity and specificity. Of all the evaluated parameters, the YS was the strongest single predictor. These findings are clinically useful for patient counseling and determining the need for closer monitoring. In fact, if these parameters are normal at 6 weeks, the pregnancy will likely continue beyond the first trimester. Although needing prospective validation, our results support changing the current standard of care of performing the first obstetric ultrasound at 9 weeks of gestation to 6 weeks of gestation. If the YS and the GS are normal, a provider can offer reassurance concerning the decreased likelihood of a pregnancy loss.
