# Author Correction: Early pregnancy ultrasound measurements and prediction of first trimester pregnancy loss: A logistic model

**DOI:** 10.1038/s41598-021-01235-0

**Published:** 2021-10-28

**Authors:** Laura Detti, Ludwig Francillon, Mary E. Christiansen, Irene Peregrin-Alvarez, Patricia J. Goedecke, Zoran Bursac, Robert A. Roman

**Affiliations:** 1 University of Tennessee Health Science Center, Division of Reproductive Endocrinology and Infertility, Department of Obstetrics and Gynecology, Memphis, TN USA; 2 Cleveland Clinic, Women’s Health Institute, Department of Obstetrics and Gynecology, Cleveland, OH USA; 3 University of Tennessee Health Science Center, Division of Biostatistics, Department of Preventive Medicine, Memphis, TN USA; 4 Florida International University, Department of Biostatistics, Miami, FL USA

Correction to: *Scientific Reports* 10.1038/s41598-020-58114-3, published online 31 January 2020

The original version of this Article contained an error in the spelling of the author Patricia J. Goedecke which was incorrectly given as Patricia J. Goeske.

The original Article has been corrected.

